# In This Issue

**DOI:** 10.1002/cfg.257

**Published:** 2003-02


**Comparative and Functional Genomics**
*Comp Funct Genom* 2003; **4**: 2–3.
Published online in Wiley InterScience (www.interscience.wiley.com). **DOI:** 10.1002/cfg.257

### In this issue

### Research Paper

Peter Giles *et al.* present a relational database for pathogenic fungi genomic and EST sequence and demonstrate its utility by using it to analyse amino acid biosynthetic pathways using the mix of complete and incomplete gene inventories currently available.

### Meeting Review: PSI

Sandra Orchard and colleagues report on the first meeting of the HUPO Proteomics Standards Initiative, which included discussions on standards for protein–protein interactions and mass spectrometry.

### Meeting Review: ECCB 2002

Clare Sansom brings us the highlights of the first European Conference on Computational Biology, held in Saarbrücken, Germany, in October 2002.

### Special section of selected reviews and papers from the 'From Genome to Life' international summer school

### Conference Reviews

Beatrice Demontera outlines the ethical issues relating to cloned and transgenic animals, highlighting a genomic approach that could provide a better understanding of the identity of these animals.

John Allen discusses the existing theories as to why chloroplasts and mitochondria have genomes, then makes the case for his co-location for redox regulation (CORR) hypothesis.

### Conference Papers

Buschlen *et al.* present a microarray study of HAP2 and HAP4 yeast mutants, to discover which genes (in particular of those encoding mitochondrial proteins) are regulated by these two genes that are known to be involved in the switch from fermentative to respiratory metabolism.

Manal Dayem and colleagues describe their microarray analysis of gene expression in wounded human keratinocytes, to study the molecular events triggered in the early stages of the wound healing process.

Sujay Chattopadhyay and colleagues report on their comparative analysis of base composition pressures in the protein coding genes of a range of Archaea.

### Special section of selected reviews and papers from the ESF program on 'Integrated Approaches to Functional Genomics' workshop on 'Ontology for Biology'

Luca Bernardi and co-organizers report on the ESF funded workshop on Ontology for Biology, which brought together computer scientists, computer linguists, philosophers and biologists.

### Conference Reviews

Evelyn Camon and colleagues present the Gene Ontology (GO) annotation project (GOA), in which proteins in the SWISS-PROT, TrEMBL, InterPro and Ensembl databases are being annotated with GO terms.

Christian Blaschke and Alfonso Valencia present an automated classification of protein function from the literature, based on statistical information extraction techniques.

Alexa McCray describes the construction of an upper-level ontology, called the UMLS semantic network, for the National Library of Medicine's Unified Medical Language System (UMLS).

Esther Ratsch and co-workers discuss their efforts towards developing a protein interaction ontology, with an initial focus on signal transduction pathways.

Copyright © 2003 John Wiley & Sons, Ltd.

Jennifer Williams and William Andersen report on their first considerations for a project to convert the GO into a formalized ontology.

In their **conference paper**, Udo Hahn and colleagues describe their work on ontology engineering from informal thesauri, with a project to build formalized ontologies from sections of the UMLS.

Steffen Staab reviews the semantic web and what it could do for biologists, highlighting three recent projects that are moving us towards this goal.

Aldo Gangemi describes the work of the Laboratory for Applied Ontology (Ontolab), discussing potential methods (and tools that they provide) for building domain ontologies.

Daniel Von Wachter describes the first stage of his work towards a medical ontology, which involves the proposal of a new philosophical theory of causation, which should help ontology engineering.

### Special section of selected reviews from the 'Standards and Ontologies for Functional Genomics' conference

Midori Harris and Helen Parkinson bring us a report from this conference, which had sessions on 'Model organism ontologies', 'Ontologies for chemistry, toxicology and other domains', 'Implementation and use of ontologies' and 'Tools for building ontologies'.

Raymond Lee and Paul Sternberg illustrate the rich knowledge available on *Caenorhabditis elegans* and describe the construction of a cell and anatomy ontology for this nematode.

Christian Stoeckert and Helen Parkinson describe the MGED ontology, which is designed as a framework for describing microarray experiments.

Robert Stevens and colleagues provide an introduction to description logics for beginners, and describe their ontology language, DAML + OIL, and editor, OilEd.

### Special section of selected articles from the ESF program on 'Integrated Approaches to Functional Genomics' workshop on 'Modelling Molecular Networks'

Paulino Gomez-Puertas and Alfonso Valencia bring us a report from this meeting, which had sessions on 'Combining theoretical and experimental approaches', 'Networks and computer simulation' and 'Assembling the puzzle'.

### Conference Paper

Javier Herrero and colleagues present their approach, which finds time-lagged correlations in gene expression from time series microarray data and uses this to build clusters of genes into a regulatory network.

### Conference Review

Hans Westerhoff discusses the current state of modelling whole cells and how this area of biology relates to physics and its modelling of systems.

Copyright © 2003 John Wiley & Sons, Ltd.

